# Identification and characterisation of the *Volvox carteri* Moco carrier protein

**DOI:** 10.1042/BSR20202351

**Published:** 2020-11-24

**Authors:** Thomas W. Hercher, Joern Krausze, Jing Yang, Martin L. Kirk, Tobias Kruse

**Affiliations:** 1 TU Braunschweig, Institute of Plant Biology, Spielmannstrasse 7, 38106 Braunschweig, Germany; 2 Department of Chemistry and Chemical Biology, The University of New Mexico, MSC03 2060, 1 University of New Mexico, Albuquerque, NM 87131-0001, U.S.A.

**Keywords:** Molybdenum cofactor, prosthetic group insertion, prosthetic group transfer

## Abstract

The molybdenum cofactor (Moco) is a redox active prosthetic group found in the active site of Moco-dependent enzymes (Mo-enzymes). As Moco and its intermediates are highly sensitive towards oxidative damage, these are believed to be permanently protein bound during synthesis and upon maturation. As a major component of the plant Moco transfer and storage system, proteins have been identified that are capable of Moco binding and release but do not possess Moco-dependent enzymatic activities. The first protein found to possess these properties was the Moco carrier protein (MCP) from the green alga *Chlamydomonas reinhardtii*. Here, we describe the identification and biochemical characterisation of the *Volvox carteri* (*V. carteri*) MCP and, for the first time, employ a comparative analysis to elucidate the principles behind MCP Moco binding. Doing so identified a sequence region of low homology amongst the existing MCPs, which we showed to be essential for Moco binding to *V. carteri* MCP.

## Introduction

The molybdenum cofactor (Moco) biosynthesis pathway is evolutionarily conserved and comprises a number of enzymatically catalysed reactions (e.g. see [1]). Within this pathway, GTP is initially converted into cyclic pyranopterin monophosphate (cPMP), which is the most stable intermediate [2]. cPMP is subsequently converted into molybdopterin (MPT), which is substrate of the molybdenum insertase (Mo-insertase [3]). MPT binds to the Mo-insertase G-domain where it is converted into adenylated MPT (MPT-AMP [4]). MPT-AMP is subsequently used as substrate for the Mo-insertase E-domain-catalysed molybdate insertion reaction [5]. This step of the Moco biosynthesis pathway is the last step that remains to be understood mechanistically, and recent work [6–8] has already laid the basis for the elucidation of the metal insertion reaction. Upon maturation, the highly oxidation-sensitive Moco must be allocated to and inserted into respective user enzymes. The most likely candidate proteins for the cellular Moco transfer system have been best characterised for the alga *Chlamydomonas reinhardtii* (*C. reinhardtii* [9–13]) and the higher plant *Arabidopsis thaliana* (*A. thaliana*, [14,15]). While *Arabidopsis* was found to harbour numerous proteins involved in Moco binding and transfer [14], *C. reinhardtii* possesses only two such proteins with different Moco binding properties [16]. Biochemical studies using recombinant proteins have identified the *C. reinhardtii* Moco carrier protein (MCP) and the *A. thaliana* Moco binding proteins (MoBP) to possess varying Moco binding properties, which were attributed to their proposed cellular functions, i.e. Moco allocation (MoBPs) and Moco storage (MCP) respectively [13,14,16]. After Moco synthesis and transfer, the cofactor must be inserted into the Moco-dependent enzymes (Mo-enzymes). In prokaryotes, a protein complex comprising enzymes involved in the final steps of Moco maturation is assumed to donate Moco to the Mo-enzymes [17] and contrary to eukaryotes, various enzyme-specific Moco chaperons have been identified here (for an overview, see [18]). In marked contrast, as yet, no Mo-enzyme specific chaperone has been identified in a eukaryote.

In studies using fully defined *in vitro* systems, the recombinant Moco-free *Neurospora crassa* (*N. crassa*) nitrate reductase (NR) [19] and *A. thaliana* sulphite oxidase proteins [5] revealed that Moco insertion into eukaryotic Mo-enzymes does not mandatorily require a Moco chaperon. However, at least for the *N. crassa* NR unknown factor(s) may be required to obtain NR with stoichiometric amounts of bound Moco [19]. Interactions of the Mo-insertase and the Moco transfer system but also the user enzymes have been described [14,15], suggesting that during [20] but also downstream [15] synthesis, Moco and its metabolites require protected transport. In this work, we describe the identification and biochemical characterisation of the *Volvox carteri* (*V. carteri*) MCP, revealing that reversible Moco binding essentially depends on a species-specific highly flexible loop that covers the accepted MCP Moco binding site.

## Experimental procedures

### Identification of *C. reinhardtii* MCP homologous proteins

To identify eukaryotic homologues to *C. reinhardtii* MCP, the *C. reinhardtii* MCP amino acid sequence (AAK77219 [12]) was used as query in pBLAST [21] searches.

### Cloning of *V. carteri* MCP

The coding sequence of *V. carteri* MCP (XP_002954772.1) was synthesised and subcloned via *Bam*HI/*Spe*I into a modified pQE80 (Quiagen, Germany) vector which allowed the expression of a C-terminal StrepII-tag^®^ fusion protein. Generation of the vector was carried out by replacing the His6 tag encoding sequence and the multiple cloning site of the pQE80 vector for the synthesised DNA sequence (5′-GAATTCATTAAAGAGGAGAAATTAACTATGTCTAGAGGATCCAGATCTCACGTGACTAGTGGCGGTTCCGCCTGGTCCCACCCCCAGTTCGAGAAGTAATGAAGCG CTAAGCTT-3′) by using the restriction sites *Eco*RI and *Hind*III. For generation of the *V. carteri* MCP low homology region (LHR) exchange variant, the following DNA sequence was synthesised: (5′-GAATTCATTAAAGAGGAGAAATTAACTATGTCTAGAGGATCCATGGCTCCACGCAAACCGATCATTGGCGTTATGGGACCAGGCGAACAGGCTACGCCTACGGATGTGG AACTGGCCACCGAACTGGGCAAGCAGATTGCGAGCCATGACTGGATTCTGCTCACAGGTGGGCGTAGTCTGGGCGTGATGGATGCGGCATGC-3′) and used to replace a DNA stretch within the *V. carteri* MCP expression construct by using the restriction sites *Eco*RI and *Spe*I. Doing so resulted in the exchange of the *V. carteri* MCP LHR region (spanning amino acids 15–26 for the *Rippkaea orientalis* (*R. orientalis*) MCP LHR region (spanning amino acids 13–19, [22]).

### Expression and purification of *V. carteri* MCP

For recombinant protein production of *V. carteri* MCP and its derivative MCP_Ro LHR_, *Escherichia coli* strains TP1000 [23], RK5206 [24] or RK5204 [24,25] were used. Expression was carried out in LB medium containing 100 µg/mL ampicillin and at 25°C. When detailed as such, 1 mM sodium molybdate was used as a supplement to the media. MCP expression was induced with 50 µM isopropyl β-d-1-thiogalactopyranoside when the optical density of the culture reached 0.1 at 600 nm. Subsequently, cells were allowed to grow aerobically for 20 h. Upon harvesting, cell lysis was performed using an Avestin Emulsiflex C5 homogenizer. A subsequent centrifugation step (24,400 × g, 60 min, 4°C) was used to clear the lysate, which was treated with DNaseI (Roche) prior to being loaded on to Strep-Tactin^®^ Superflow^®^ high capacity resin (IBA). All following affinity purification steps were carried out according to the manufacturer (IBA) description and at 4°C. Purity of the eluted proteins was routinely documented by SDS/PAGE analysis. 12% SDS/PAGE gels were TGX-stained and processed using ImageLab 6.0 with a ChemiDoc XRS+ System (Bio-Rad). Pure protein fractions were concentrated (Vivaspin 6, Sartorius AG) and used directly for the subsequent biochemical characterisation. For the determination of its oligomeric state, *V. carteri* MCP was subjected to gel filtration experiments with a Superdex 200 10/300 Increase column (GE Healthcare) in a buffer containing 100 mM Tris-HCl pH 8.0, 150 mM NaCl, and 5% (v/v) glycerol. The column was calibrated with a High Molecular Weight calibration kit (GE Healthcare).

### Quantification of protein concentrations

Protein concentration determination was carried out using the Pierce™ BCA Protein-Assay (Thermo Scientific). Absorption at 562 nm was measured using a Thermo Scientific™ Multiskan™ GO Microplate Spectrophotometer.

### Thermal-shift experiments using recombinant *V. carteri* MCP

Thermal shift (Thermo Fluor) assays were employed to determine the thermal stability of *V. carteri* MCP. Using a CFX96/C1000 qPCR Instrument (Bio-Rad) melt curves were generated with SYPRO^®^ Orange Protein Gel Stain (Sigma) and analysed using the CFX Manager Software V 3.0 (Bio-Rad). Melting temperatures were determined for the specified buffer conditions. To obtain MCP samples with different Moco saturations, an MCP protein preparation possessing a Moco occupancy of 0.24 ± 0.03 was diluted with corresponding amounts of Moco/MPT-free MCP.

### *V. carteri* MCP Moco stabilisation

Pure recombinant *V. carteri* MCP protein obtained by expression and purification from *E. coli* strain TP1000 (Moco/MPT saturation ∼ 0.21) was incubated at 4, 22 and 60°C for 160 min. At defined time intervals, protein equivalent to 2–20 pmol of Moco/MPT (inferred from T_0 min_) was oxidised (*n*=3) to allow its quantification via the stable oxidation product FormA. Protein concentration was routinely determined for every sample at the end of the timed experiment (T_160 min_).

### Quantification of *V. carteri* MCP-bound Moco/MPT

Quantification of *V. carteri* MCP and MCP_Ro LHR_-bound Moco/MPT was carried out via FormA-based HPLC analysis, essentially as described in [8] and using synthetic FormA as calibration standard [26]. A total of 0.25–2.5 µg of pure protein were oxidised to quantify Moco/MPT amounts of the TP1000 purified protein. In the case of Moco-free protein (obtained from strain RK5204), up to 50 µg were oxidised to document that no trace amounts of Moco/MPT were present.

### UV VIS spectroscopy

For UV VIS spectroscopy, recombinant *V. carteri* MCP was adjusted to 600 µM in purification buffer (100 mM Tris-HCl pH 8.0, 150 mM NaCl, 1 mM EDTA, 5% (v/v) glycerol). Spectra were recorded using a PerkinElmer UV/VIS Spectrometer Lambda 25 between 380 and 620 nm.

### Inductively coupled plasma mass spectrometry

Molybdenum content in purified protein samples was quantified with an Agilent 7700 Series ICP-MS (Agilent Technologies) using a standard calibration curve of inorganic molybdenum (Fluka). The molybdenum calibration for the inductively coupled plasma mass spectrometry (ICP-MS) was in the range of 0.1–20 µg/l. Samples were mixed with rhodium (Rh(NO_3_)_3_) as an internal standard. Gathered data were processed using MassHunter work station software. Protein samples with concentrations of 0.8–1.5 mmol/l were used for analysis. Samples were diluted 1:1 in 69% Suprapur^®^ nitric acid (Merck), after an overnight incubation at room temperature they were diluted with Milli-Q water prior to ICP-MS measurements.

### Nit-1 assay

The *nit-1* assay was conducted largely as described previously [22]. Reconstitution was conducted overnight on ice. The NR reaction was allowed to proceed for 30 min. Formed nitrite was quantified at 540 nm [22,27,28] using a Thermo Scientific™ Multiskan™ GO Microplate Spectrophotometer.

### One-to-one threading

The construction of a *V. carteri* MCP model was carried out with the one-to-one threading option of the Phyre2 modelling server [29] with the structures of the two confirmed MCPs from *C. reinhardtii* ([13], PDB: 2IZ6, 74.6% sequence identity with *V. carteri* MCP) and *R. orientalis* ([22], PDB: 6Y01, 51.5% sequence identity with *V. carteri* MCP) serving as templates. Phyre2 was run using a local alignment method and with secondary structure scoring enabled using a secondary structure weighing of 0.1.

### Small-angle X-ray scattering

Small-angle X-ray scattering (SAXS) experiments were conducted on beamline BM29 of the European Synchrotron Radiation Facility, Grenoble, France [30]. A concentration of 3.9 g/l of gel filtration purified *V. carteri* MCP were loaded into a quartz capillary and subjected to irradiation with X-rays of 12,500 eV at room temperature. The scattering intensities were recorded within a momentum transfer range of 1.0 × 10^−2^ to 5.0 × 10^−1^ Å^−1^ using a PILATUS 1 M hybrid photon-counting detector. A total of ten scattering images were collected with an exposure time of 0.5 s per image. The scattering data were processed with the ATSAS suite [31]. We created oligomeric models of *V. carteri* MCP by arranging the Phyre2 model that was based on the *C. reinhardtii* MCP structure similar to the arrangements found in the *C. reinhardtii* MCP crystal structure ([13], PDB: 2IZ6) resulting in a *V. carteri* MCP monomer A, a dimer AB, a dimer AD and a tetramer ABCD. We finally calculated theoretical scattering curves of these oligomeric models and compared them with the experimental scattering curve with the program CRYSOL [32].

## Results

### Identification of *C. reinhardtii* MCP homologous proteins

**Figure 1:**
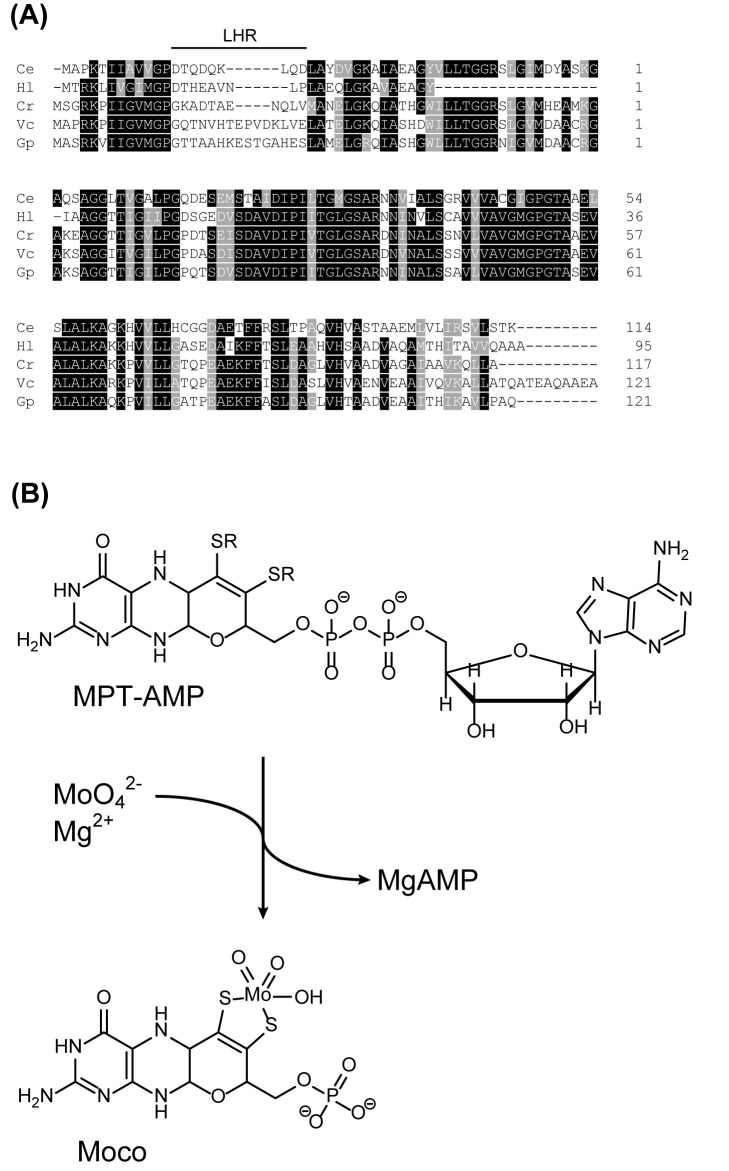
Identification of the *V. carteri* MCP (**A**) Amino acid sequence alignment of MCPs from *C. eustigma* (Ce, GAX76684.1), *H. lacustris* (Hl, GFH18826.1), *C. reinhardtii* (Cr, XP_001694446.1), *V. carteri* (Vc, XP_002954772.1) and *G. pectorale* (Gp, KXZ54074.1). Strictly conserved residues are highlighted in black and conserved residues are highlighted in grey. A low homology region (LHR) identified within the compared sequences is indicated. (**B**) Reaction scheme of Mo-insertase catalysed Moco formation [3], [5]. The chemical structures of MPT-AMP and Moco are shown. The alignment shown in (A) was generated with Clustal Omega.

Hitherto a single MCP (from the green alga *C. reinhardtii*) has been characterised biochemically in depth [9–13], which documented its function for reversible Moco (Figure 1B) binding. Amongst eukaryotes, pBLAST [21] searches revealed three other potential MCP homologues, all within the order *Chlamydomonadales*. Here potential MCP homologues have been identified in the algae *Chlamydomonas eustigma, Haematococcus lacustris, Gonium pectorale* and *V. carteri* (Figure 1A). Amongst these, the putative MCP from *V. carteri* was chosen for subsequent characterisation as a rapid phylogenetic analysis [33] revealed this to be the closest relative to *C. reinhardtii* MCP.

### The oligomeric state of *V. carteri* MCP

**Figure 2:**
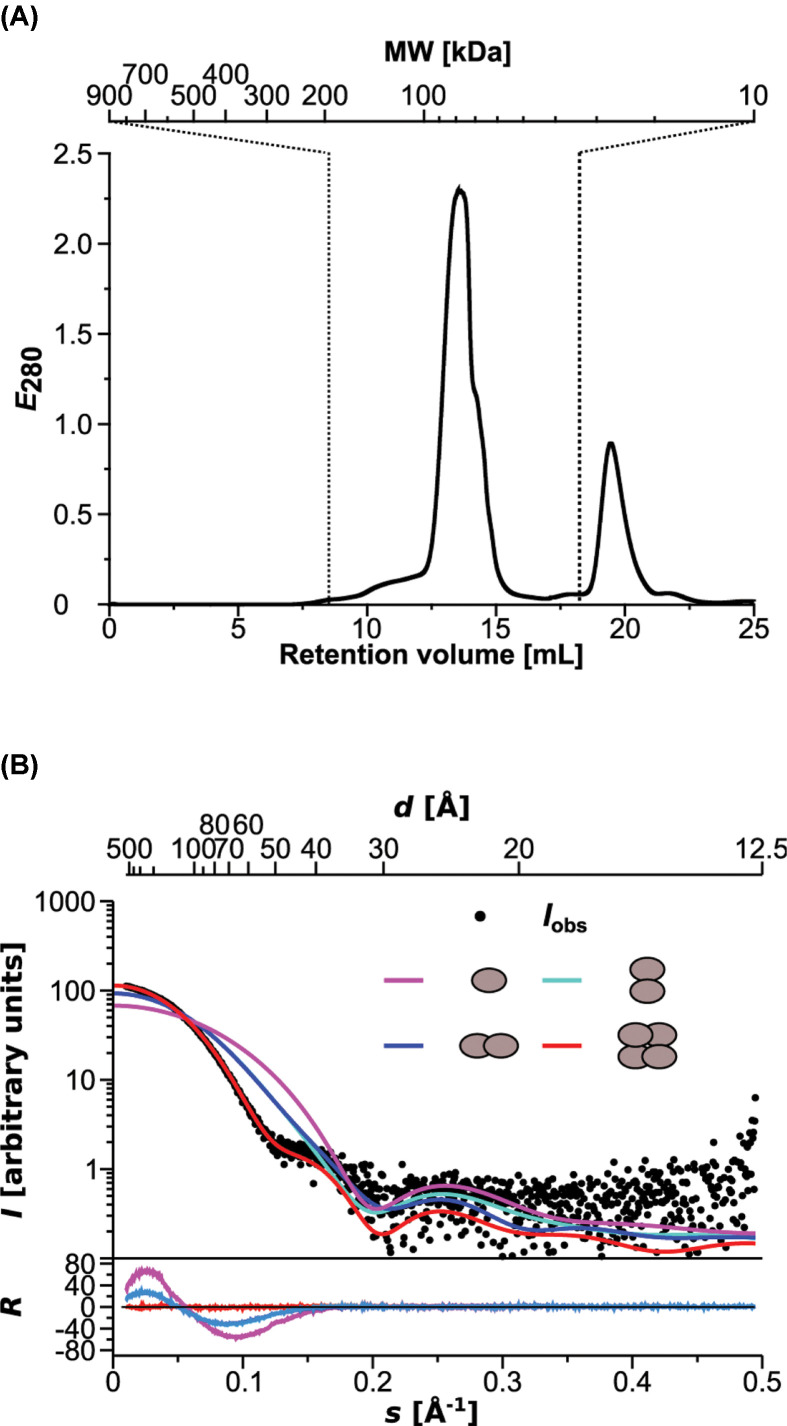
Oligomeric state of the *V. carteri* MCP (**A**) Gel filtration elution profile, absorbance at 280 nm plotted vs retention volume (axis of abscissa) and corresponding molecular weight (alternate axis of abscissa). The retention volume of the *V. carte*ri MCP peak (∼13 ml) corresponds to a molecular weight of ∼80 kDa, indicative for a tetramer. The dashed lines indicate the upper and lower exclusion limits of the column. (**B**) Experimental SAXS intensities (•) and theoretical intensities plotted against momentum transfer (axis of abscissa) and corresponding resolution (alternate axis of abscissa) for momomer (magenta), dimer AB (blue), dimer AD (cyan) and tetramer (red). The plot of the residual (R = (I_obs_ − I_calc_)/σI_obs_) represents the quality of fit, which is highest for the tetramer (also indicated by χ^2^ = 1.96).

The oligomeric state of *V. carteri* MCP was determined by gel filtration and SAXS. In gel filtration, *V. carteri* MCP migrates at a retention volume of ∼13 ml, which correlates with an apparent molecular weight of ∼80 kDa (Figure 2A). Considering the monomeric molecular weight of the *V. carteri* MCP construct that was used in the present study (∼19.5 kDa), the gel filtration strongly suggests a *V. carteri* MCP tetramer in solution, agreeing with the tetrameric states that were published for *C. reinhardtii* MCP [13] and *R. orientalis* MCP [22]. SAXS confirmed that notion of a *V. carteri* MCP tetramer. SAXS further revealed that the arrangement of the *V. carteri* MCP tetramer obeys *D*_2_ symmetry, as a *V. carteri* MCP tetramer model based on the structure of the *C. reinhardtii* MCP *D*_2_ tetramer shows the best agreement with the experimental SAXS curve derived from *V. carteri* MCP in solution (Figure 2B).

### Biochemical characterisation of *V. carteri* MCP

**Figure 3:**
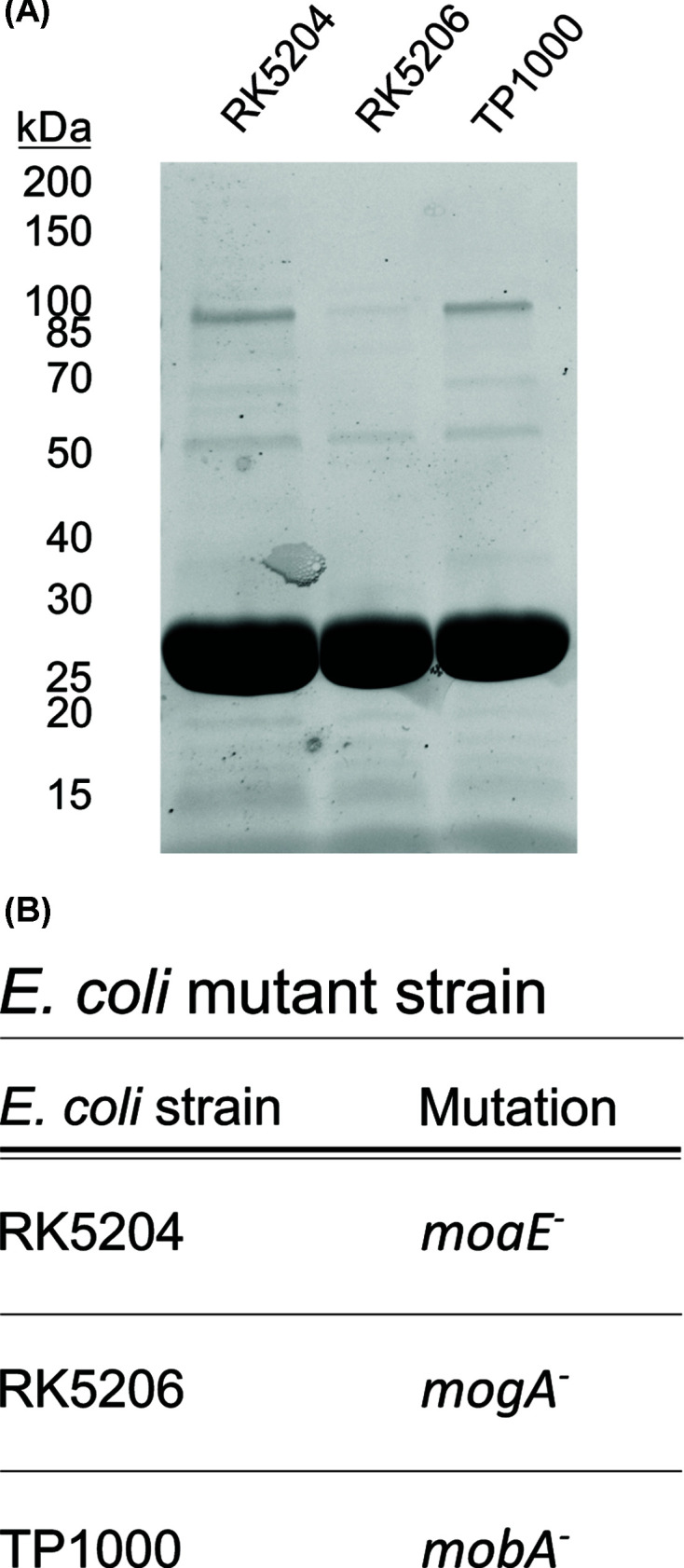
Purification of the recombinant *V. carteri* MCP from different *E. coli* expression strains (**A**) TGX-stained SDS PA gel exemplarily documenting the purity of recombinant *V. carteri* MCP preparations expressed and purified from the indicated strains. (**B**) Genotype of the *V. carteri* MCP expression strains [23–25] used for recombinant protein production documented in (A).

**Figure 4:**
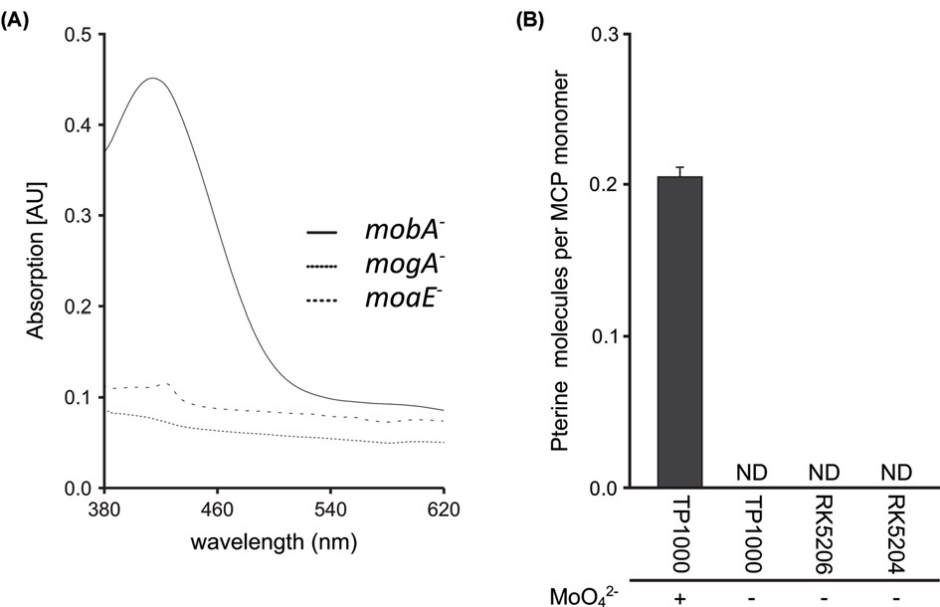
Biochemical characterisation of the *V. carteri* MCP (**A**) Spectral properties of recombinant *V. carteri* MCP. The spectra shown was derived from 600 µM recombinant *V. carteri* MCP solutions obtained from proteins expressed and purified from the *mobA*^−^ strain TP1000 [23], the MPT-accumulating *mogA*^−^ strain RK5206 [24] and the Moco/MPT-free *moaA*^−^ strain RK5204 [24,25]. (**B**) Co-purified Moco/MPT with recombinant *V. carteri* MCP. Recombinant expression and purification were carried out in the Moco containing host strain TP1000 (cultivated with and without supplementing media with 1 mM molybdate) [13], the MPT accumulating host strain RK5206 and the Moco/MPT free host strain RK5204. Bars represent the standard deviation, resulting from three full replicas. Abbreviation: ND, not detectable.

Recombinant expression and purification of the putative MCP from *V. carteri* was carried out in the Moco accumulating *E. coli* strain TP1000 [23], the MPT accumulating *E. coli* strain RK5206 [24] and the Moco/MPT free *E. coli* strain RK5204 (Figure 3A,B [24,25]). This yielded recombinant protein preparations of high purity as documented by SDS/PAGE analysis (Figure 3A). UV-VIS spectroscopic data for the recombinant putative MCP from *V. carteri* (Figure 4A) document overall comparable spectral properties of the putative MCP from *V. carteri* and the described MCP from *C. reinhardtii* [13]. Consistent with previous reports, we have identified an absorption band at ∼420 nm (23,800 cm^−1^) that we tentatively assign as a dithiolene → Mo charge transfer transition (Figure 4A [13,34–37]). Electronic absorption data for [(bdt)MoO_3_]^2−^ [38] (bdt = benzene-1,2-dithiol) and [(bdt)MoO_2_(OSiPh_3_)]^1−^ [39], which are small molecule Moco analogues, indicate that there are no electronic transitions at wavelengths above 325 nm (30,800 cm^−1^) for [(bdt)MoO_3_]^2−^. Thus, the *V. carteri* MCP absorption data suggest that at least one of the oxo ligands is likely to be protonated. For example, the di-oxo compound [(bdt)MoO_2_(OSiPh_3_)]^1−^ possesses lower energy electronic absorption bands at ∼395 nm (25,300 cm^−1^) and ∼495 nm (20,200 cm^−1^) [39]. The protonation of a Moco oxo ligand is likely to be extremely important in providing a labile, presumably aqua, ligand that can dissociate and allow for amino acid coordination to Mo when the cofactor is inserted into apo-proteins.

### Quantitative biochemistry of recombinant *V. carteri* MCP

We proceeded to quantify the amount of protein bound Moco/MPT via HPLC-based FormA analysis, which revealed a Moco/MPT binding stoichiometry of ∼0.21 ± 0.01 for recombinant *V. carteri* MCP (Figure 4B). The metal-free Moco precursor MPT was not co-purified with recombinant *V. carteri* MCP as documented by quantitative FormA analysis of *V. carteri* MCP derived from the Moco free but MPT accumulating strain RK5206 (Figure 4B). To give additional evidence for *V. carteri* MCP Moco selectivity, we carried out an ICP-MS analysis. In accordance with our hypothesis, Mo amounts above the experimental/methodical error range defined where only detected in *V. carteri* MCP preparations derived from strain TP1000 (Figure 4B and Supplementary Figure S1). Therefore, we concluded, that *V. carteri* MCP binds Moco but not MPT. As observed previously for *C. reinhardtii* MCP [13], when no molybdate was supplemented to the expression medium no Moco was detected bound to MCP (Figure 4B).

### *V. carteri* MCP Moco stabilising properties

**Figure 5:**
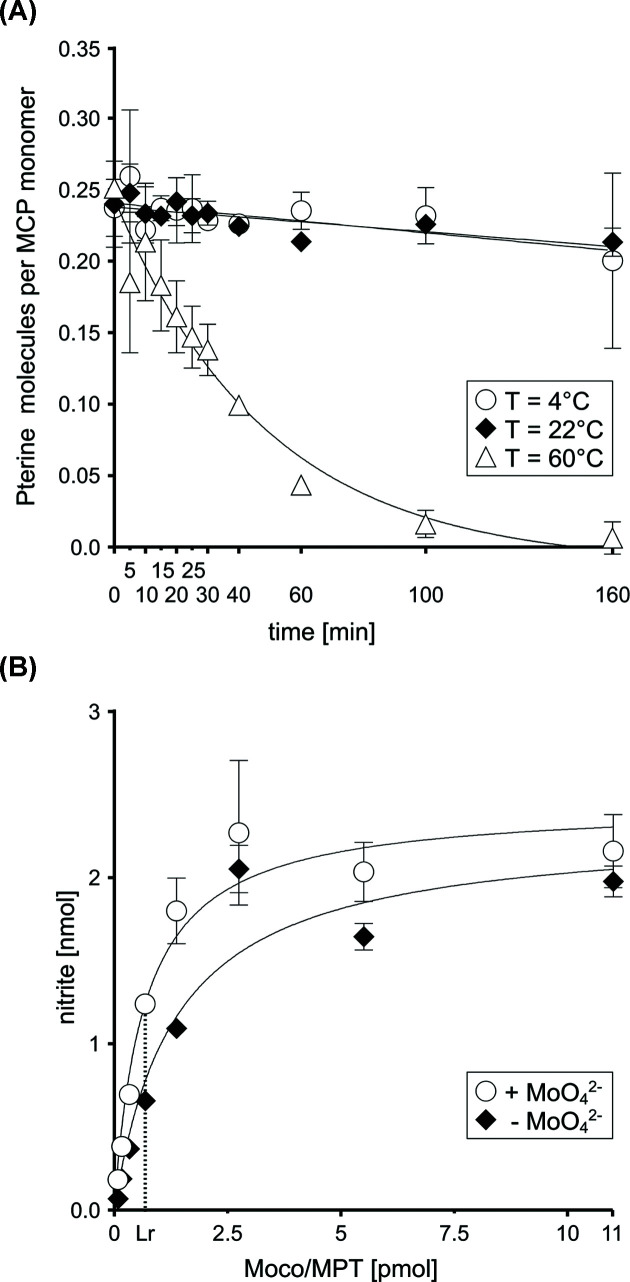
Moco stabilising properties of the *V. carteri* MCP (**A**) The *V. carteri* MCP was incubated at three different temperatures and for 160 min. At indicated time points, Moco/MPT contents were quantified HPLC FormA based (*n*=3). (**B**) Results of *nit-1* assay conducted with *V. carteri* MCP in the presence (white circles) or absence (black diamonds) of 1 mM sodium molybdate. Non-linear curve fitting was carried out using the GraphPad Prism software version 6.02. The results are described in amounts of nitrite produced over the course of the experiment (30 min). Standard deviations are the result of three independent experiments. The linear reconstitution range was found to span 0.09–0.690 pmol Moco (8–64 ng *V. carteri* MCP, with R^2^ = 0.9839 for when molybdate was supplemented and R^2^ = 0.9888 without). Abbreviation: Lr, linear range.

The stabilisation of Moco by *V. carteri* MCP was surveyed. To this end, the protein purified from *E. coli* TP1000 [23] and hence saturated with Moco (∼0.21 ± 0.01, Figure 4B) was incubated at different temperatures (4, 22 and 60°C) for 160 min. Determination of the protein concentration revealed that, in accordance with thermal shift assay results (Supplementary Figure S2 and Table S1), there was no loss in the MCP amounts over the course of 160 min regardless of the incubation temperature. Furthermore, FormA quantification revealed that there was barely any loss in Moco amounts at 4°C and 22°C whereas at 60°C *V. carteri* MCP-bound Moco diminished to 0.00 over the course of 160 min (Figure 5A).

### Functional complementation of the *N. crassa* NR using recombinant *V. carteri* MCP

To provide evidence that *V. carteri* MCP is fully capable of donating Moco to user enzymes, we carried out *nit-1* assay [28] to qualitatively detect physiologically active Moco. This revealed that *V. carteri* MCP is fully capable of donating Moco to the Moco-free NR present in *N. crassa nit-1* strain crude cell extracts [28] as documented by the fact that Moco-dependent enzymatic activity is reconstituted successfully (Figure 5B). Furthermore, the successful reconstitution even without the addition of molybdate proves that significant amounts of physiologically active Moco is bound to the protein.

### Moco binding to *V. carteri* MCP

**Figure 6:**
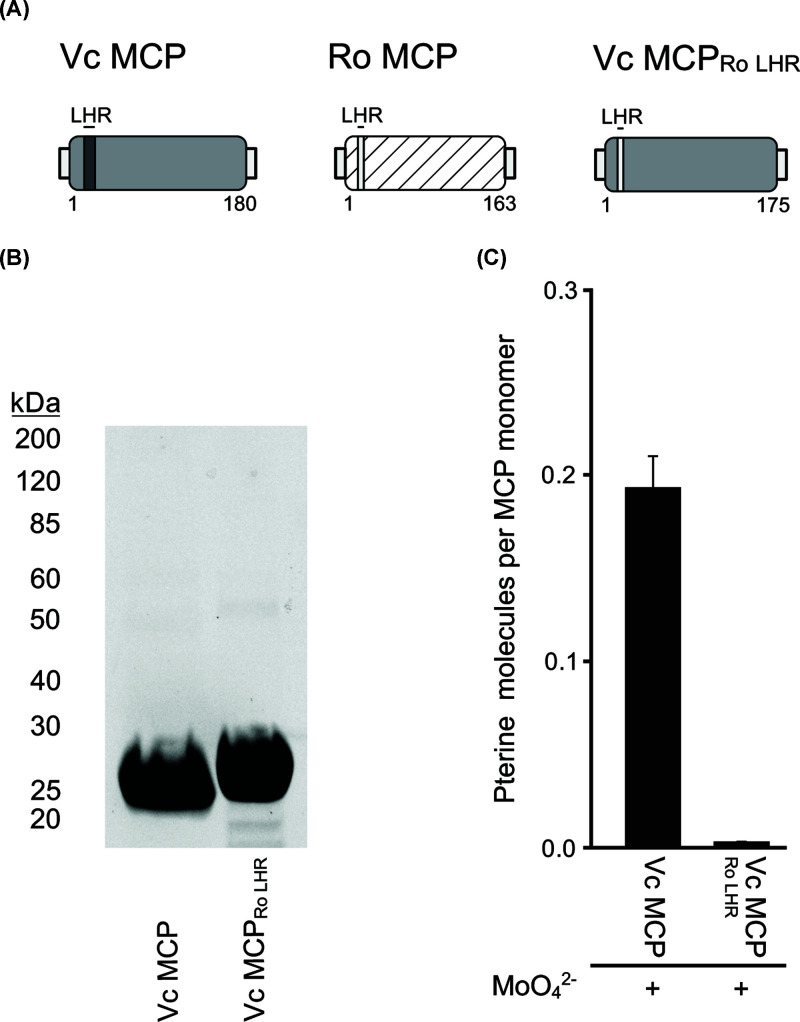
Characterisation of the chimeric *V. carteri* MCP variant Vc MCP_Ro LHR_ (**A**) Schematic representation of *V. carteri* MCP (VcMCP) and the *Rippkaea orientalis* MCP (RoMCP). The first and last amino acids are indicated. LHR is indicated by a box. (**B**) TGX stained, representative SDS PA gel documenting the homogeneity of VcMCP and VcMCP_Ro LHR_ protein preparations. (**C**) Co-purified Moco/MPT with recombinant VcMCP and the variant VcMCP_Ro LHR_. Recombinant expression and purification was carried out in the Moco containing host strain TP1000 [23], cultivated in media supplemented with 1 mM sodium molybdate. Bars represent the standard deviation, resulting from three full replicas.

The amino acid sequence comparison (Figure 1A) of eukaryotic MCPs revealed a region of low sequence homology (LHR) located in the N-terminal part of the protein sequence. Results of docking experiments published for *C. reinhardtii* MCP [13] as well as a bacterial MCP from *R. orientalis* [22] indicate that Moco binding probably occurs in a predominantly positively charged crevice that is located in proximity to the LHR. We therefore investigated the LHR’s role by creating a chimeric *V. carteri* MCP, which had its endogenous LHR switched for its counterpart from *R. orientalis* MCP (Figure 6A). Earlier reports indicated that recombinant *R. orientalis* MCP purified *from E. coli* (Figure 6B) has a significantly lower saturation with Moco [22] than what we report here for *V. carteri* MCP. Accordingly, compared with wildtype *V. carteri* MCP (Figure 6C), the chimera showed a significantly reduced Moco saturation (∼0.03, Figure 6C), that was comparable with the saturation reported for *R. orientalis* MCP [22].

## Discussion

Hitherto, the cellular Moco transfer system has been fundamentally characterised in the higher plant *A. thaliana* [14,15] and in the green alga *C. reinhardtii* [9–13]. Progress in understanding how Moco is transferred from source to sink involved the characterisation of *in vivo* protein–protein interactions [15] which were complemented by the biochemical characterisation of MCP and MoBP proteins respectively [13,14], though as yet it is not known how Moco binding and transfer to the cognate user enzymes are implemented on the structural level. Progress in genome sequencing and annotation has allowed us to identify, as of yet, uncharacterised eukaryotic *C. reinhardtii* MCP homologues, from which one identified in the alga *V. carteri* was characterised within the present study.

**Figure 7:**
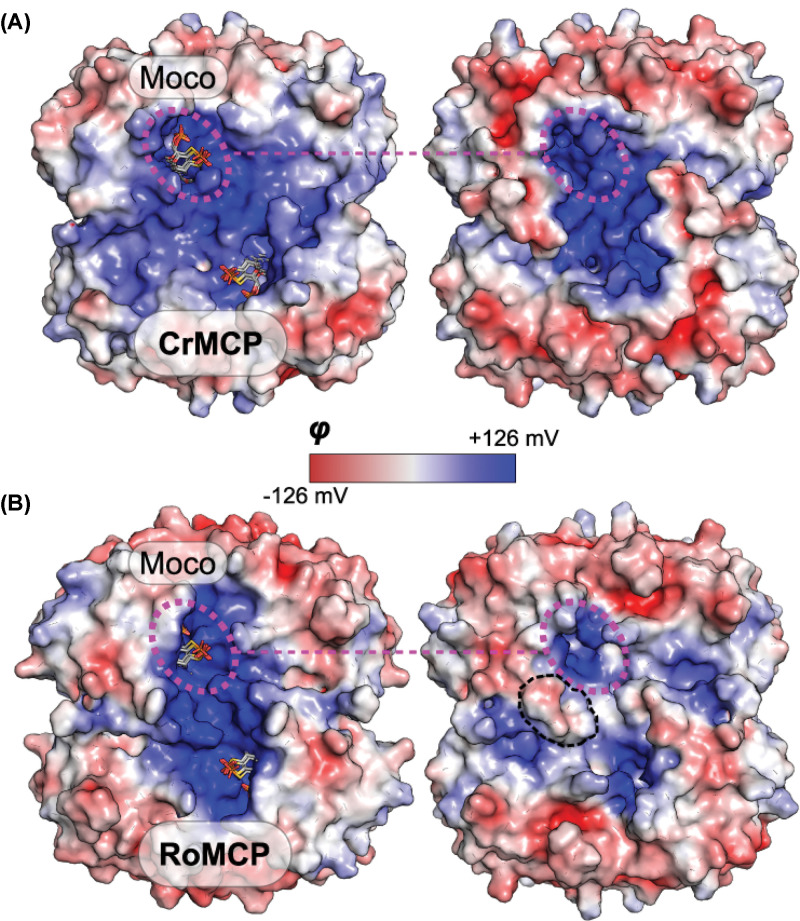
Proposed Moco binding site of *V. carteri* MCP Surface representation of different MCP structures and models coloured according to their surface potential ranging from ≤ −126 mV (red) to ≥ +126 mV (blue). (**A**) Left, the *C. reinhardtii* MCP (CrMCP) structure published earlier [13] with the top five results of Moco docking calculations redone according to the specifications by Fischer *et al.* [13]. Right, one-to-one threading model of the *V. carteri* MCP (VcMCP) based on the CrMCP structure. (**B**) Left, the *Rippkaea orientalis* (*R. orientalis*) MCP (RoMCP) structure and the Moco docking results published recently [22]. Right, one-to-one threading model of VcMCP based on the RoMCP structure. The area bounded by a black broken line indicates a small region, the amino acid side chains of which are oriented differently than in the model shown in panel A. The positive patch visible in panel A is present but hidden from sight underneath these side chains.

As observed for *C. reinhardtii* MCP [13] also *V. carteri* MCP was found to bind Moco selectively over the metal-free Moco precursor MPT which was shown by the HPLC-based detection of the pterin backbone as well as by the ICP-MS based detection of the Moco molybdenum component. Consistent results were obtained from the *nit-1* based reconstitution and the Moco stabilisation assay and hence we conclude that *V. carteri* MCP possesses overall comparable characteristics to *C. reinhardtii* MCP [13]. *V. carteri* MCP is the second eukaryotic MCP described and given the fact, that MCPs share overall high levels of sequence and structural similarities (this work, [13,22]), we assume that these have also a conserved Moco binding site. To verify this hypothesis for *V. carteri* MCP, we carried out one-to-one threading using the Phyre2 server [29] to create two models based on the structural templates *C. reinhardtii* MCP [13] and *R. orientalis* MCP (the first structurally resolved prokaryotic MCP [22]) respectively. Our analysis showed that both models have a surface charge distribution featuring a positively charged area in a crevice, which was already earlier suggested to be the *C. reinhardtii* MCP Moco binding site [13] (Figure 7). Further we identified a flexible loop of low sequence homology located in close proximity to the putative Moco binding site which we found to be of crucial importance for the *V. carteri* MCP Moco binding capability. As a possible explanation for the low sequence conservation between the loops from various MCPs, we suggest that these are important for the adaption of the MCPs to the existent cellular contexts in the respective host organisms. Consistently, we suggest that this loop may possess a sequence independent lid function.

Our combined data hence confirm that the crevice is the Moco binding site common for pro- and eukaryotic MCPs and the LHR loop is essential for Moco binding, probably because it acts as a lid for the Moco binding site.

## Supplementary Material

Supplementary Figures S1-S2 and Table S1

## Data Availability

The data and material presented in this manuscript are available from the corresponding author on reasonable request.

## Abbreviations

bdt: benzene-1,2-dithiol
cPMP: cyclic pyranopterin monophosphate
ICP-MS: inductively coupled plasma mass spectrometry
LHR: low homology region
MCP: Moco carrier protein
MoBP: Moco binding protein
Moco: molybdenum cofactor
Mo-enzyme: Moco-dependent enzyme
Mo-insertase: molybdenum insertase
MPT: molybdopterin
MPT-AMP: adenylated MPT
NR: nitrate reductase
SAXS: small-angle X-ray scattering
UV: ultraviolet
VISV: visible
