# Supplementary material for: Identification and characterisation of the Volvox carteri Moco carrier protein
Source: Biosci Rep. 2020 Nov 24;40(11):BSR20202351. doi: 10.1042/BSR20202351 (PMC7687042; doi:10.1042/BSR20202351)
Supplement: Supplementary Figures S1-S2 and Table S1 [file BSR-2020-2351_supp.pdf]

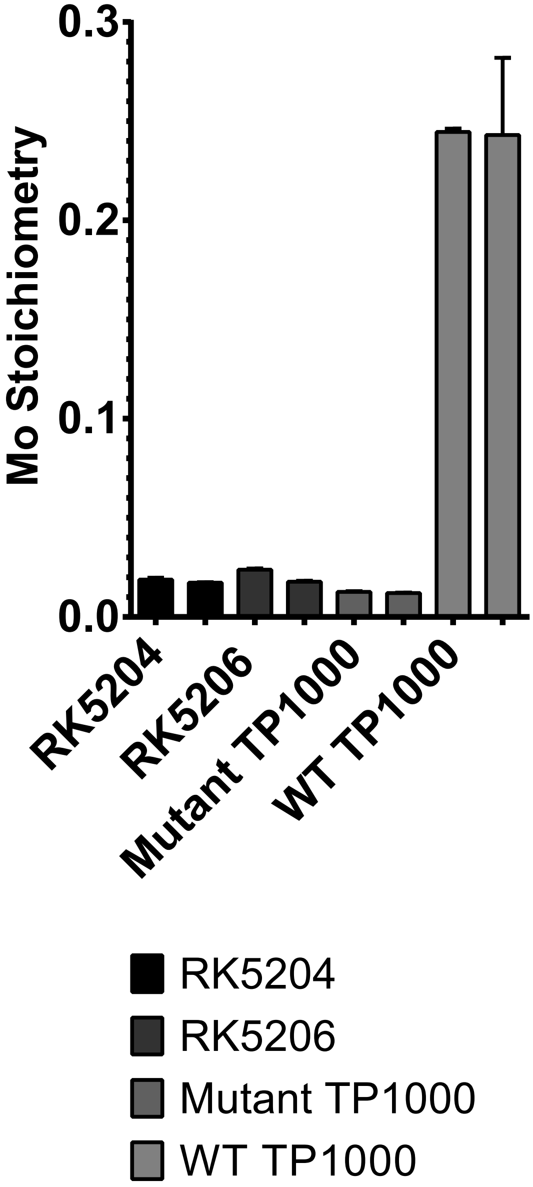

0.3
0.2
0.1
0.0
Mo Stoichiometry
RK5204
RK5206
Mutant TP1000
WT TP1000
RK5204
RK5206
Mutant TP1000
WT TP1000

Figure -S1-

Figure S1: ICP-MS based analysis of *Volvoxcarteri* Moco carrier proteins. Recombinant wildtype (WT) *Volvoxcarteri*Moco carrier protein(VcMCP) and the recombinant chimeric VcMCP protein variant $VcMCP_{Ro\ LHR}$ (Mutant, Figure 6) were analyzed for Mo content after expression and purification from*E. coli* strains RK5204, RK5206 and TP1000 respectively (see Figure 4). The Mo-binding stoichiometry defined for VcMCP preparations obtained from the Moco/MPT free strain RK5204 documents the Mo background arising from either methodical or experimental errors.

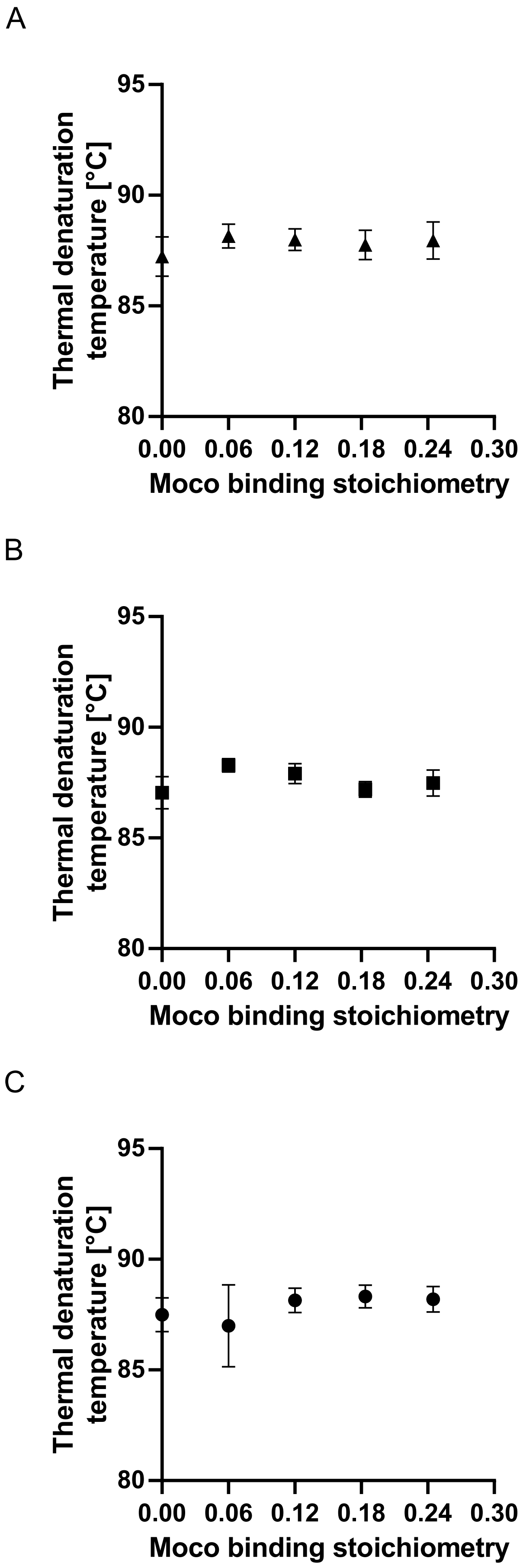
A
Thermal denaturation temperature [°C]
95
90
85
80
0.00
0.06
0.12
0.18
0.24
0.30
Moco binding stoichiometry
B
Thermal denaturation temperature [°C]
95
90
85
80
0.00
0.06
0.12
0.18
0.24
0.30
Moco binding stoichiometry
C
Thermal denaturation temperature [°C]
95
90
85
80
0.00
0.06
0.12
0.18
0.24
0.30
Moco binding stoichiometry

Figure -S2-

Figure S2: Thermal shift assays of *Volvoxcarteri* Moco carrier protein with varying Moco occupancies. Pictured are the results of three independent thermal shift assays conducted with three different buffer conditions. In each condition the*Volvoxcarteri*(*V. carteri*) Moco carrier protein (MCP) was analyzed with Moco binding stoichiometriesranging from 0.00 to 0.24 +/- 0.03. Buffer condition (A) 200mM Tris-HCl, pH 8.0, 7.5 mM NaCl. Buffer condition (B) 200 mM MES-KOH, pH 5.5. Buffer condition. In (C) *V. carteri*MCP was analyzed in 7.5 mM NaCl in ddH_2O. The standard deviations shown was obtained from three full replicates.

Table S1: Thermal shift assay of Moco free *Volvoxcarteri*Moco carrier protein. Displayed in the table are the result of a thermal shift assay conducted with the *Volvoxcarteri*Moco carrier protein purified from the molybdenum cofactor and molybdopterin free *E. coli* strain RK5204[24, 25]. Tested buffer conditions and melting temperatures are displayed beside each other.

	Condition	Melt Temp °C
1	H2O	14
2	200 mM Sodium phosphate citrate pH 4.5	12.5
3	200 mM Sodium phosphate citrate pH 5.0	None
4	200 mM MES pH 5.5	79.75
5	200 mM MES pH 6.0	80.25
6	200 mM MES pH 6.5	89.5
7	200 mM HEPES pH 7.0	86.5
8	200 mM HEPES pH 7.5	87.75
9	200 mM HEPES pH 8.0	87.5
10	200 mM Tris-HCl pH 8.0	87.5
11	200 mM Tris-HCl pH 8.5	89.5
12	200 mM Tris-HCl pH 9.0	81.5
13	H2O 75 mM NaCl	89.5
14	200 mM Sodium phosphate citrate 75 mM NaCl pH 4.5	None
15	200 mM Sodium phosphate citrate 75 mM NaCl pH 5.0	None
16	200 mM MES 75 mM NaCl pH 5.5	86.0
17	200 mM MES 75 mM NaCl pH 6.0	86.25
18	200 mM MES 75 mM NaCl pH 6.5	83.75
19	200 mM HEPES 75 mM NaCl pH 7.0	89.5
20	200 mM HEPES 75 mM NaCl pH 7.5	85.25
21	200 mM HEPES 75 mM NaCl pH 8.0	87.5
22	200 mM Tris-HCl 75 mM NaCl pH 8.0	85.25
23	200 mM Tris-HCl 75 mM NaCl pH 8.5	89.5
24	200 mM Tris-HCl 75 mM NaCl pH 9.0	13.0
25	H2O 150 mM NaCl	89.5
26	200 mM Sodium phosphate citrate 150 mM NaCl pH 4.5	None
27	200 mM Sodium phosphate citrate 150 mM NaCl pH 5.0	None
28	200 mM MES 150 mM NaCl pH 5.5	None
29	200 mM MES 150 mM NaCl pH 6.0	86.5
30	200 mM MES 150 mM NaCl pH 6.5	None
31	200 mM HEPES 150 mM NaCl pH 7.0	86.5
32	200 mM HEPES 150 mM NaCl pH 7.5	85.75
33	200 mM HEPES 150 mM NaCl pH 8.0	89.5
34	200 mM Tris-HCl 150 mM NaCl pH 8.0	None
35	200 mM Tris-HCl 150 mM NaCl pH 8.5	89.5
36	200 mM Tris-HCl 150 mM NaCl pH 9.0	89.5
37	H2O 225 mM NaCl	89.5
38	200 mM Sodium phosphate citrate 225 mM NaCl pH 4.5	None
39	200 mM Sodium phosphate citrate 225 mM NaCl pH 5.0	None
40	200 mM MES 225 mM NaCl pH 5.5	89.5
41	200 mM MES 225 mM NaCl pH 6.0	None
42	200 mM MES 225 mM NaCl pH 6.5	None
43	200 mM HEPES 225 mM NaCl pH 7.0	89.5
44	200 mM HEPES 225 mM NaCl pH 7.5	87.0
45	200 mM HEPES 225 mM NaCl pH 8.0	87.0
46	200 mM Tris-HCl 225 mM NaCl pH 8.0	None
47	200 mM Tris-HCl 225 mM NaCl pH 8.5	89.5
48	200 mM Tris-HCl 225 mM NaCl pH 9.0	12.5
49	H2O 300 mM NaCl	14.0

50	200 mM Sodium phosphate citrate 300 mM NaCl pH 4.5	None
51	200 mM Sodium phosphate citrate 300 mM NaCl pH 5.0	None
52	200 mM MES 300 mM NaCl pH 5.5	None
53	200 mM MES 300 mM NaCl pH 6.0	None
54	200 mM MES 300 mM NaCl pH 6.5	None
55	200 mM HEPES 300 mM NaCl pH 7.0	89.5
56	200 mM HEPES 300 mM NaCl pH 7.5	89.5
57	200 mM HEPES 300 mM NaCl pH 8.0	89.5
58	200 mM Tris-HCl 300 mM NaCl pH 8.0	89.5
59	200 mM Tris-HCl 300 mM NaCl pH 8.5	89.5
60	200 mM Tris-HCl 300 mM NaCl pH 9.0	None
61	H2O 375 mM NaCl	12.5
62	200 mM Sodium phosphate citrate 375 mM NaCl pH 4.5	None
63	200 mM Sodium phosphate citrate 375 mM NaCl pH 5.0	None
64	200 mM MES 375 mM NaCl pH 5.5	None
65	200 mM MES 375 mM NaCl pH 6.0	None
66	200 mM MES 375 mM NaCl pH 6.5	None
67	200 mM HEPES 375 mM NaCl pH 7.0	89.5
68	200 mM HEPES 375 mM NaCl pH 7.5	89.5
69	200 mM HEPES 375 mM NaCl pH 8.0	89.5
70	200 mM Tris-HCl 375 mM NaCl pH 8.0	None
71	200 mM Tris-HCl 375 mM NaCl pH 8.5	89.5
72	200 mM Tris-HCl 375 mM NaCl pH 9.0	89.5
73	H2O 450 mM NaCl	12.5
74	200 mM Sodium phosphate citrate 450 mM NaCl pH 4.5	None
75	200 mM Sodium phosphate citrate 450 mM NaCl pH 5.0	None
76	200 mM MES 450 mM NaCl pH 5.5	None
77	200 mM MES 450 mM NaCl pH 6.0	None
78	200 mM MES 450 mM NaCl pH 6.5	None
79	200 mM HEPES 450 mM NaCl pH 7.0	None
80	200 mM HEPES 450 mM NaCl pH 7.5	None
81	200 mM HEPES 450 mM NaCl pH 8.0	None
82	200 mM Tris-HCl 450 mM NaCl pH 8.0	89.5
83	200 mM Tris-HCl 450 mM NaCl pH 8.5	89.5
84	200 mM Tris-HCl 450 mM NaCl pH 9.0	89.5
85	H2O 525 mM NaCl	None
86	200 mM Sodium phosphate citrate 525 mM NaCl pH 4.5	None
87	200 mM Sodium phosphate citrate 525 mM NaCl pH 5.0	None
88	200 mM MES 525 mM NaCl pH 5.5	None
89	200 mM MES 525 mM NaCl pH 6.0	None
90	200 mM MES 525 mM NaCl pH 6.5	None
91	200 mM HEPES 525 mM NaCl pH 7.0	None
92	200 mM HEPES 525 mM NaCl pH 7.5	71.5
93	200 mM HEPES 525 mM NaCl pH 8.0	None
94	200 mM Tris-HCl 525 mM NaCl pH 8.0	61.5
95	200 mM Tris-HCl 525 mM NaCl pH 8.5	None
96	200 mM Tris-HCl 525 mM NaCl pH 9.0	12.5

Table -S1-
